# Supplementary material for: An N4-like Caulobacter phage requires host smooth lipopolysaccharide biosynthesis for infection
Source: bioRxiv. 2025 Oct 24:2025.10.24.684376. Preprint. [Version 1] doi: 10.1101/2025.10.24.684376 (PMC12633290; doi:10.1101/2025.10.24.684376)
Supplement: 1 [file NIHPP2025.10.24.684376V1-supplement-1.pdf]

bioRxiv preprint doi: https://doi.org/10.1101/2025.10.24.684376; this version posted October 24, 2025. The copyright holder for this preprint (which was not certified by peer review) is the author/funder, who has granted bioRxiv a license to display the preprint in perpetuity. It is made available under aCC-BY 4.0 International license.

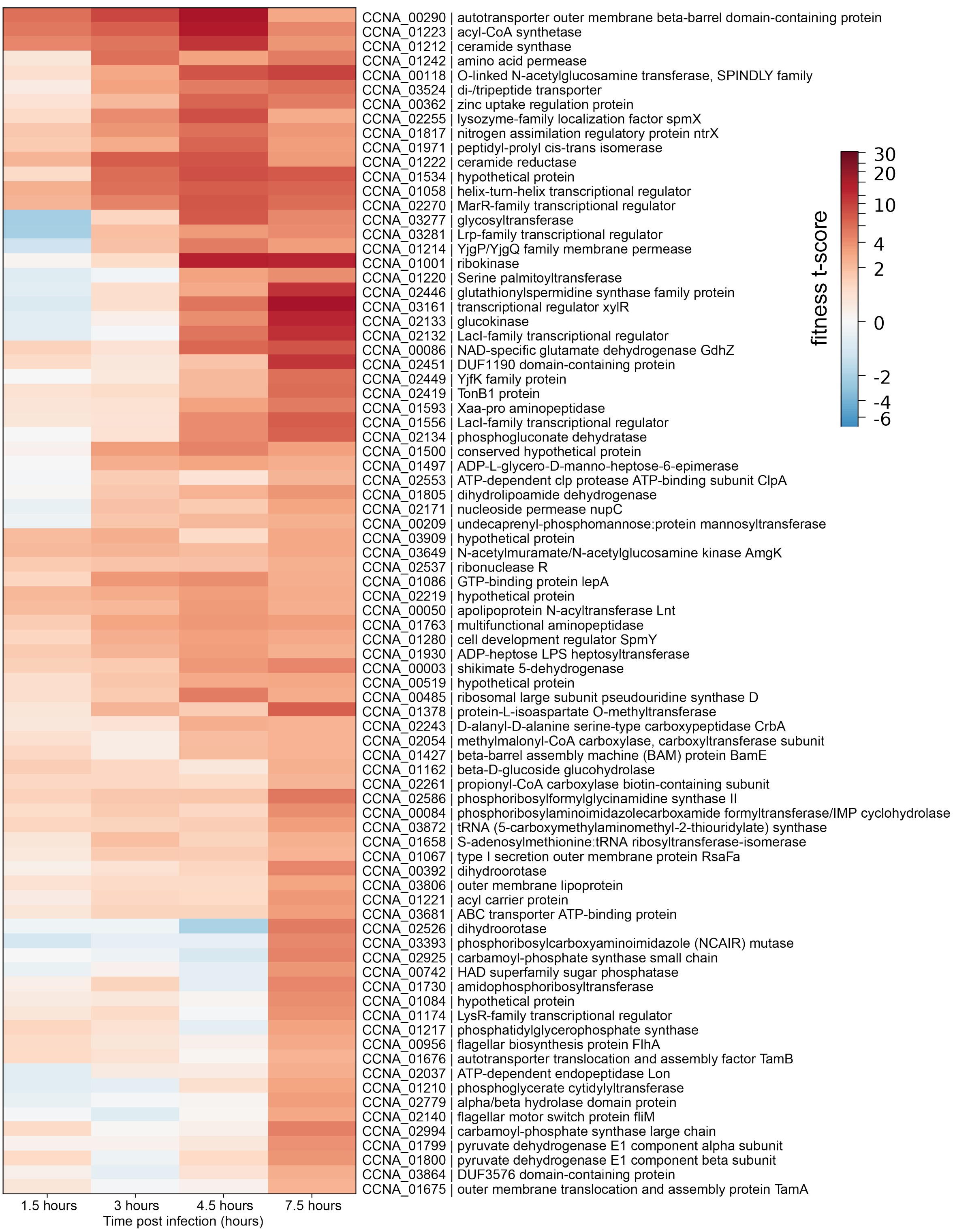

Figure S1. Hierarchically clustered heatmap of *C. crescentus* gene fitness t-scores at 1.5, 3.0, 4.5, and 7.5 h post-infection with CirceC at MOI=1. Rows include genes with $| t | \geq 4$at ≥1 time point. Figure shows clustered genes not presented in panel 7B. Complete fitness data are presented in Table S3.

bioRxiv preprint doi: https://doi.org/10.1101/2025.10.24.684376; this version posted October 24, 2025. The copyright holder for this preprint (which was not certified by peer review) is the author/funder, who has granted bioRxiv a license to display the preprint in perpetuity. It is made available under aCC-BY 4.0 International license.

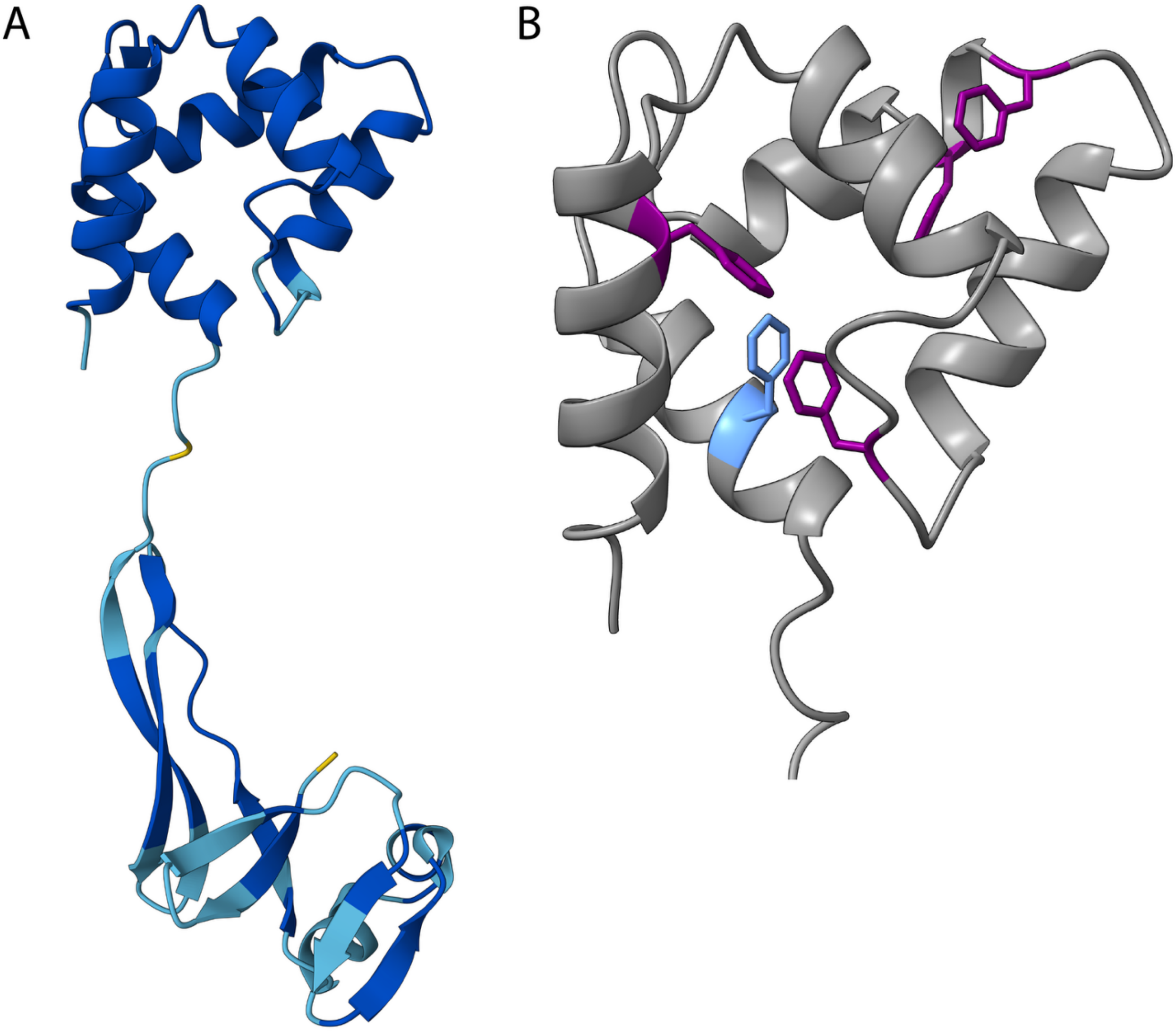

Figure S2. Predicted gp063 structure. Structure of gp063 predicted by AlphaFold3. A) Full-length gp063 colored based on pIDDT score (pTM=0.53). B) C-terminal domain of gp063. Phenylalanine residues are colored purple. Phenylalanine 91 is colored blue.
